# Renal dysfunction reduces the diagnostic and prognostic value of serum CC16 for acute respiratory distress syndrome in intensive care patients

**DOI:** 10.1186/s12890-020-01245-0

**Published:** 2020-08-12

**Authors:** Jinle Lin, Wuyuan Tao, Jian Wei, Jian Wu, Wenwu Zhang, Jianbing Ye, Xuan Fu, Shiyong Zeng, Qingli Dou, Lijun Wang, Fang Tian

**Affiliations:** 1 Department of Emergency Medicine, Affiliated Baoan Hospital of Shenzhen, Southern Medical University, 118 LongjingEr Road, Baoan, Shenzhen, 518101 Guangdong China; 2 Department of Respiratory, East Zone Sixth Division, Guangdong Provincial People’s Hospital, Guangdong Academy of Medical Science, Guangdong Provincial Geriatrics Institute, The second School of Clinical Medicine, Southern Medical University, No. 106, Zhongshan Second Road, Guangzhou, 510000 Guangdong China; 3 Department of Critical Care Medicine, Affiliated Baoan Hospital of Shenzhen, Southern Medical University, Shenzhen, 518101 Guangdong China

**Keywords:** Acute respiratory distress syndrome, Acute kidney injury, Chronic kidney injury, Biomarkers, Club cell protein 16

## Abstract

**Background:**

Contradictory results regarding changes in serum club cell protein 16 (CC16) levels in patients with acute respiratory distress syndrome (ARDS) have been reported, challenging the value of CC16 as a diagnostic and prognostic marker for ARDS. We have also observed increased serum CC16 levels in patients with renal dysfunction (RD). Therefore, the present study aimed to determine whether RD affects the diagnostic performance of CC16 for ARDS in intensive care unit (ICU) patients.

**Methods:**

We measured serum CC16 concentrations in 479 ICU patients, who were categorized into six groups according to their diagnoses: control, acute kidney injury (AKI), chronic kidney disease (CKD), ARDS, ARDS+AKI, and ARDS+CKD. The sensitivity, specificity, and cutoff values for serum CC16 were assessed by receiver operating characteristic curve analysis.

**Results:**

Serum CC16 concentrations were higher in the ARDS group than in the control group, and in ARDS patients with normal renal function, serum CC16 could identify ARDS and predict survival outcomes at 7 and 28 days. However, serum CC16 levels were similar among the ARDS+AKI, ARDS+CKD, AIK, and CKD groups. Consequently, in patients with AKI and/or CKD, the specificity of CC16 for diagnosing ARDS or ARDS+RD decreased from 86.62 to 2.82% or 81.70 to 2.12%, respectively. Consistently, the CC16 cutoff value of 11.57 ng/ml in patients with RD differed from the established values of 32.77–33.72 ng/ml with normal renal function. Moreover, the predictive value of CC16 for mortality in ARDS+RD patients was lost before 7 days but regained by 28 days.

**Conclusion:**

RD reduces the diagnostic specificity, diagnostic cutoff value, and predictive value for 7-day mortality of serum CC16 for ARDS among ICU patients.

## Background

Acute respiratory distress syndrome (ARDS) is an acute lung disease with high mortality and morbidity in intensive care units (ICUs). No effective interventions have been established for its treatment, largely because the underlying physiological processes remain unknown. Early correct diagnosis is crucial to determine effective management. However, traditional methods, including PaO_2_/FiO_2_ measurement and X-ray, as mentioned in the Berlin definition, reveal changes that lag behind the actual progression of ARDS. Consequently, more than 20 potential biomarkers have been explored for their potential value in the diagnosis and prediction of ARDS in current studies [1], including the club cell protein (CC16).

CC16 is produced by club cells and was first described by the German anatomist Max Clara in 1937 [2]. The bronchial epithelium consists of 80% club cells, such as basal or nonciliated secretory cells, particularly in the distal bronchia [3]. According to previous studies, CC16, as the most abundant secretory protein found in the surface fluids of the airways, plays an important role in the maintenance and repair of lung airways [4]. Additionally, CC16 was reported as a potential biomarker of pulmonary injury caused by inhaled ozone, chlorine, and lipopolysaccharide (LPS) [2].

Five previous studies have evaluated the dynamics of CC16 expression in ARDS patients, but the results remain controversial. First, in 2006, a prospective multicenter observational study of 78 critical care patients conducted by the Quebec Critical Care Network found that an increase in the serum CC16 level was linked to the onset of ARDS as well as negative outcomes in ARDS patients [5]. In addition, Determann et al. reported increased plasma levels of CC16 in 22 patients with ventilator-associated pneumonia who developed ARDS. They found a better diagnostic capacity of CC16 at the cutoff point of 30 ng/ml compared to surfactant Protein D, Krebs von den Lungen, and soluble receptor for advanced glycation end products. Interestingly, an increase in CC16 was seen prior to a diagnosis of ARDS [6]. Wutzler et al. further observed that increases in serum CC16 levels accompanied secondary respiratory complications in patients with multiple injuries [7]. In contrast, Kropski et al. found lower median plasma CC16 levels in ARDS patients than in patients with cardiogenic pulmonary edema (22 ng/ml vs. 55 ng/ml) [8]. Furthermore, Ware et al. indicated that lower levels of CC16 (cutoff value at 9.2 ng/ml) might help clinicians distinguish ARDS patients from sepsis patients [9]. The contradictory findings of these studies suggest that not only ARDS but also other factors influence serum CC16 levels.

Previously, we found that an increased serum CC16 level (cutoff point at ≥33.3 ng/ml) can predict the onset of ARDS and is negatively correlated with the PaO_2_/FiO_2_ ratio among ARDS patients [10]. However, we later observed that renal dysfunction (RD) separately raises the serum CC16 level. In the present study, we retrospectively evaluated whether RD interferes with the diagnostic performance of serum CC16 for ARDS in ICU patients.

## Methods

### Study population

From March 2013 to March 2015, patients admitted into our ICU were enrolled in the present study if they met the following criteria for inclusion: 1) age > 18 and < 75 years; 2) ICU stay of > 12 h; 3) blood samples collected < 6 h after admission; and 4) diagnosis was confirmed before discharge. The Institutional Human Ethics Committee of affiliated Baoan Hospital of Shenzhen, Southern Medical University approved the protocols employed in this observational study. Written informed consent was obtained from each patient or their legal guardian.

### Data collection and laboratory examination

Baseline data, including age, gender, blood pressure, body temperature, respiratory rate, heart rate, shock index, and PaO_2_/FiO_2_ ratio, were collected within 3 h after admission to the ICU. Seven-day mortality was recorded for all enrolled patients.

The levels of N-terminal of the prohormone brain natriuretic peptide (NT-proBNP), albumin, and serum creatinine were synchronously measured within 3 h after admission.

All of the above data were compiled in a Microsoft Office Excel 2003 spreadsheet (Microsoft Corp., Seattle, WA, USA) for subsequent analysis.

### Diagnosis criteria

ARDS was diagnosed according to the Berlin definition [11]: 1) acute course, < 7 days; 2) bilateral opacities consistent with pulmonary edema, as detected by computed tomography or X-ray; and 3) a PaO_2_/FiO_2_ ratio < 300 mmHg, with ventilation support (positive end expiratory pressure or continuous positive airway pressure ≥ 5 mmH_2_O).

Acute kidney injury (AKI) or chronic kidney disease (CKD) were diagnosed according to the clinical practice guidelines of the 2012 Kidney Disease Improving Global Outcomes organization [12]. AKI was defined by an increase in creatinine of ≥0.3 mg/dL (26.4 μmol/ml) within 48 h or ≥ 50% above baseline, known or presumed to have occurred within the previous 7 days. CKD was defined by an estimated glomerular filtration rate < 60 ml/min·1.73 m^2^ for > 3 months.

### Subgroup division

Two senior physicians divided the patients into six subgroups after retrospectively reviewing patients’ diagnoses based on their clinical conditions within 3 h after admission: 1) control group: ICU patients without ARDS or RD; 2) AKI group: AKI patients without ARDS; 3) CKD group: CKD patients without ARDS; 4) ARDS group: ARDS patients without RD; 5) ARDS+AKI group: ARDS patients with AKI; and 6) ARDS+CKD group: ARDS patients with CKD.

### Measurement of serum CC16

Blood samples were immediately centrifuged at 3000 rpm for 10 min, and the serum was stored at − 60 °C prior to analysis. The CC16 concentration was determined using an enzyme-linked immunosorbent assay kit (R&D Systems, Minneapolis, MN, USA) following the manufacturer’s instructions. A laboratory staff member blinded to patients’ clinical data performed each assay in duplicate.

### Statistical analysis

The data are presented as the mean ± standard deviation or median (interquartile range) as indicated. Student’s t test or Mann-Whitney U test was used for comparisons between the groups when appropriate based on the normality of the data. Categorical data were compared using the χ^2^ or Kruskal-Wallis test. Differences among more than three subgroups were assessed using one-way analysis of variance. Linear correlations among the PaO_2_/FiO_2_ ratio and serum levels of CC16, albumin, creatinine, and NT-proBNP were calculated using the Pearson linear correlation model. Receiver operating characteristic (ROC) curves were generated to assess the optimal cutoff value, sensitivity, and specificity values. A *P*-value < 0.05 was considered statistically significant. Statistical analyses were performed using the SPSS software package (version 20.0; SPSS Inc., Chicago, IL, USA). Statistical graphs were created using GraphPad Prism 3.0 software (GraphPad Software Inc., La Jolla, CA, USA).

## Results

### Patients’ baseline characteristics

A total of 479 critical care patients were recruited into our study, including 230 cases in the control group, 45 cases in the AKI group, 47 cases in the CKD group, 83 cases in the ARDS groups, 61 cases in the ARDS+AKI group, and 13 cases in the ARDS+CKD group. Lower blood pressure and higher incidence rates of pneumonia and sepsis were found in the ARDS and ARDS+AKI groups compared with the control group. However, higher blood pressure and a higher proportion of cardiogenic pulmonary edema were observed in the CKD and ARDS+CKD groups compared with the control group (Table 1). The reasons for ICU admission and outcomes of patients are described in Supplementary Tables 1 and 2. Relationship between serum CC16 levels with the severity of AKI or CKD was described in Supplementary Tables 3 and 4.

**Table 1:**
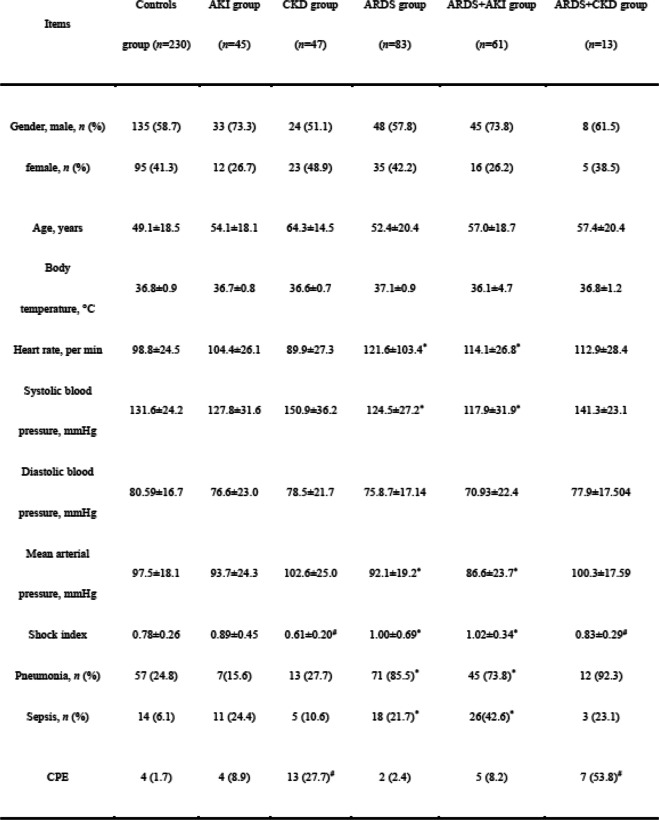
Baseline characteristics of patients at admission

Note: Data were analyzed using Student’s t test or χ^2^ test. AKI, acute kidney injury; *CKD* Chronic kidney disease, *ARDS* Acute respiratory distress syndrome, *CPE* Cardiogenic pulmonary edema

*P < 0.05 between the ARDS group or ARDS+AKI group and the control group; #*P* < 0.05 between the CKD group or ARDS+CKD group and the control group

### Serum CC16 levels in six subgroups

**Fig. 1:**
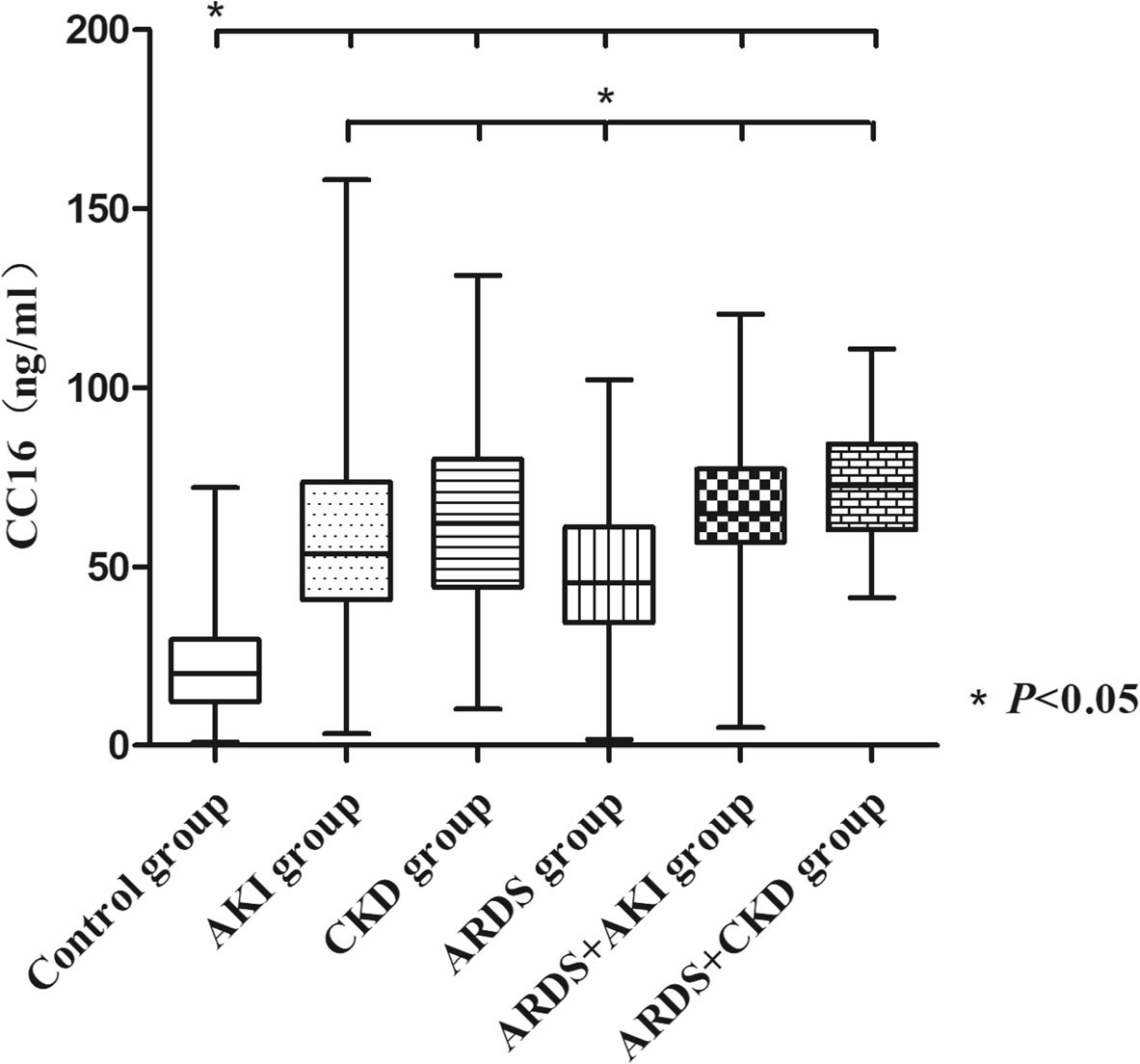
Comparison of serum CC16 levels in the control group, AKI group, CKD group, ARDS group, ARDS+AKI group, and ARDS+CKD group. *P < 0.05

The serum CC16 concentration was higher in the ARDS group than in the control group (47.78 ± 19.92 ng/ml vs. 22.23 ± 13.28 ng/ml, *P* = 0.001). Even higher serum CC16 concentrations were observed in the following groups, with no significant differences among these groups: ARDS+AKI group (64.89 ± 20.47 ng/ml), ARDS+CKD group (72.21 ± 18.63 ng/ml), AKI group (59.77 ± 26.76 ng/ml), and CKD group (62.77 ± 25.11 ng/ml) (Fig. 1).

### ROC curves for the diagnostic value of serum CC16 level in ARDS

**Fig. 2:**
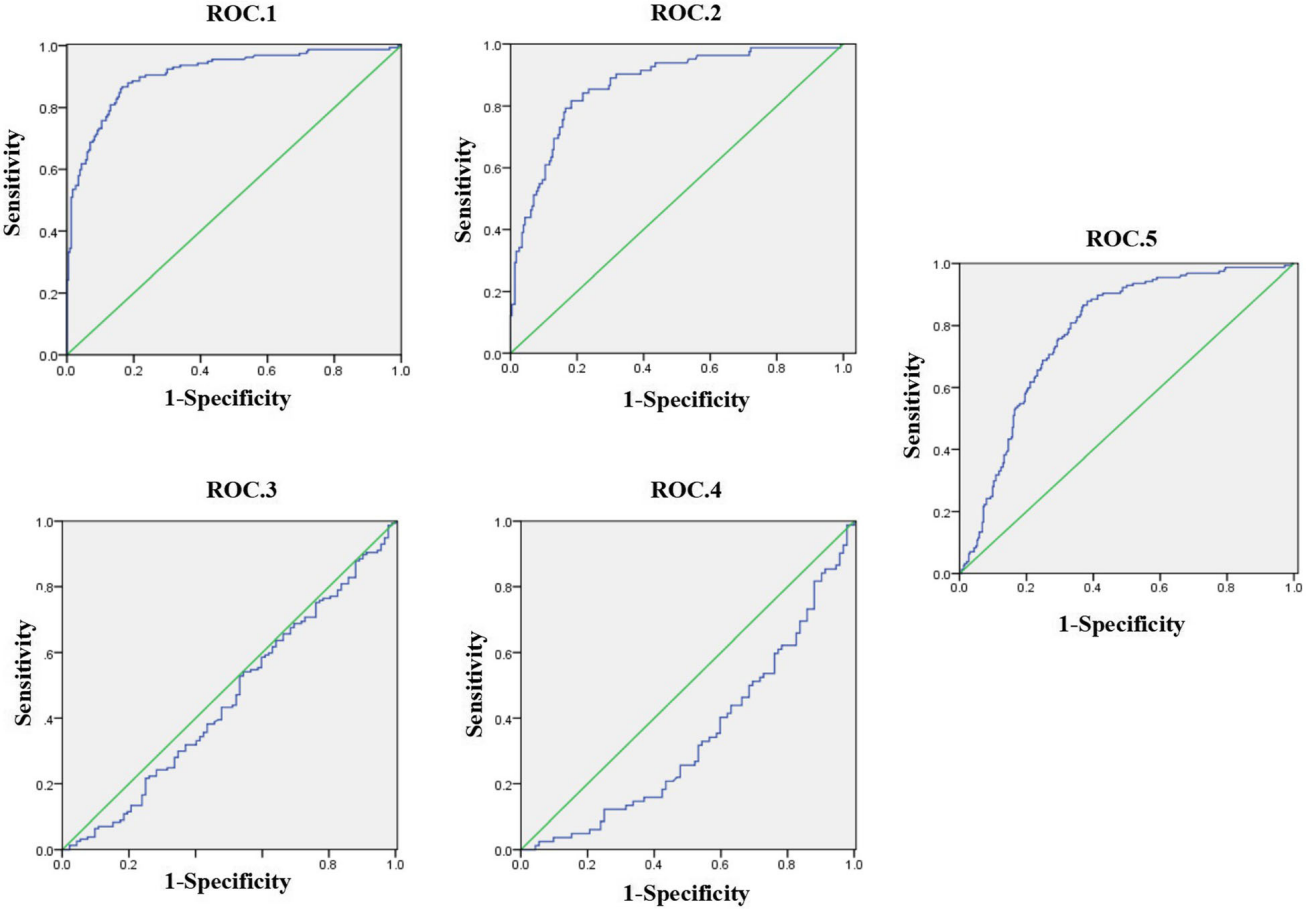
Five ROC curves for the value of serum CC16 in diagnosing ARDS in critical care patients: ROC.1 shows the ability of serum CC16 to distinguish patients in the ARDS group vs. the control group; ROC.2 shows the ability of serum CC16 to distinguish patients in the ARDS group, ARDS+AKD group, and ARDS+CKD group vs. the control group; ROC.3 shows the ability of serum CC16 to distinguish patients in the ARDS group vs. the AKI group and CKD group; ROC.4 shows the ability of serum CC16 to distinguish patients in the ARDS group, ARDS+AKD group, and ARDS+CKD group vs. the AKI group and CKD group; and ROC.5 shows the ability of serum CC16 to distinguish patients among all groups

We constructed ROC curves to evaluate the diagnostic performance of serum CC16 in the different groups of critical care patients (Table 2 and Fig. 2). Although the ROC curves for different groups showed a similar sensitivity, with RD as a baseline characteristic, the specificity for distinguishing ARDS from ARDS with a renal condition (AKI or CKD) decreased from 86.62 to 2.82% (ROC.1 vs. ROC.3) or 81.70 to 2.12% (ROC.2 vs. ROC.4). Consistently, a different cutoff value of 11.57 ng/ml was found, which was lower than the previous values of 32.77 ng/ml and 33.72 ng/ml.

**Table 2:**
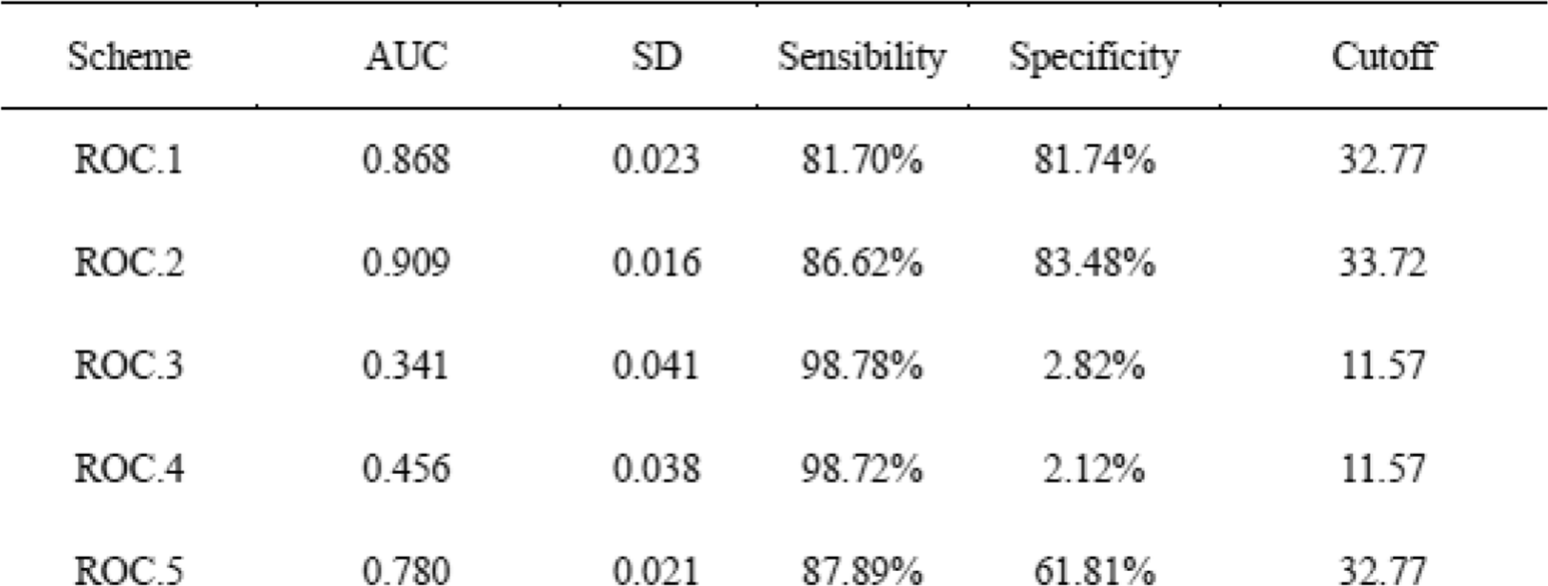
Receiver operating characteristic (ROC) curves for the diagnostic value of serum club cell protein 16 (CC16) level for acute respiratory distress syndrome (ARDS) in different schemes

Notes: *ROC* Receiver operating characteristic, *AUC* Area under the curve, *SD* Standard deviation, *AKI* Acute kidney injury, *CKD* Chronic kidney disease, *ARDS* Acute respiratory distress syndrome. *P < 0.05

ROC.1: ARDS group vs. control group;

ROC.2: ARDS group, ARDS+AKD group, and ARDS+CKD group vs. control group;

ROC.3: ARDS group vs. AKI group and CKD group;

ROC.4: ARDS group, ARDS+AKD group, and ARDS+CKD group vs. AKI group and CKD group;

ROC.5: ARDS group, ARDS+AKD group and ARDS+CKD group vs. control group, AKI group and CKD group

### Correlation between serum CC16 levels and other clinical parameters

**Fig. 3:**
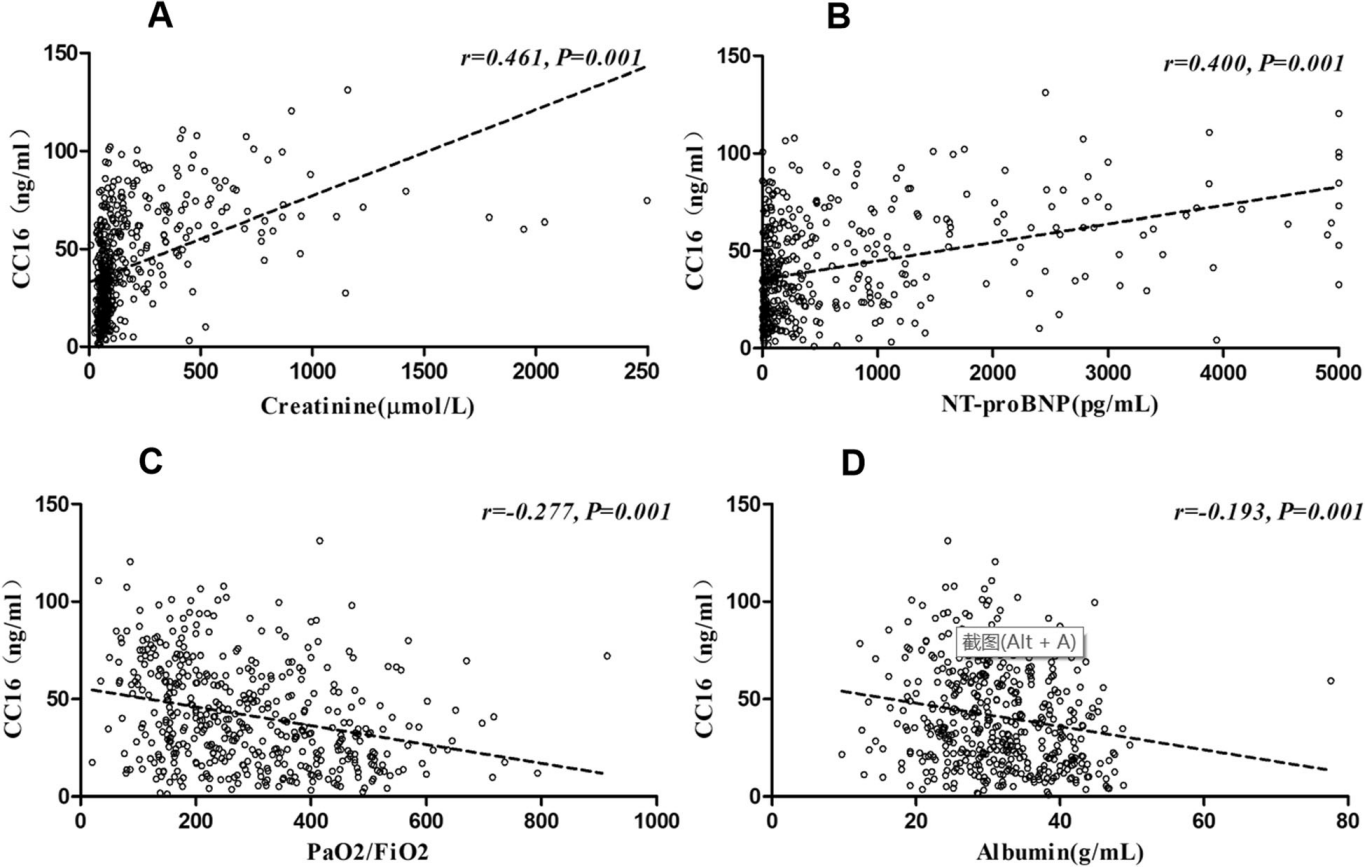
Correlations of serum CC16 level and other clinical parameters. **a** A positive correlation was observed between the serum CC16 level and serum creatinine level in all groups. **b** A positive correlation was observed between the serum CC16 level and NT-proBNP level in all groups; **c** A negative correlation was observed between the serum CC16 level and PaO_2_/FiO_2_ ratio in all groups. **d** A negative correlation was observed between the serum CC16 level and albumin level in all groups

Compared to the control group, the ARDS group had a higher concentration of serum CC16 and lower values for the PaO_2_/FiO_2_ ratio and albumin. However, decreased renal function in the AKI, CKD, ARDS+AKI, and ARDS+CKD groups was consistently related to increased serum levels of CC16, NT-proBNP, and creatinine compared to those in the control and ARDS groups. Furthermore, Pearson correlation analysis showed that the serum CC16 level were positively correlated with the serum creatinine (*r* = 0.461, Fig. 3a) and NT-proBNP (*r* = 0.400, Fig. 3b) levels but negatively correlated with the PaO_2_/FiO_2_ ratio (*r* = 0.-277, Fig. 3c) and albumin level (*r* = − 0.193, Fig. 3d).

### Relationship between serum CC16 level and outcomes in ICU patients

Increased serum CC16 levels were observed among non-surviving patients in all groups, in relation to 7-day mortality (68 cases, 14.19%) and 28-day mortality (121 cases, 25.26%) compared with the serum CC16 levels in the surviving patients of each group (54.99 ± 25.74 ng/ml vs. 38.57 ± 25.76 ng/ml for 7-day mortality; 51.01 ± 25.89* ng/ml vs. 37.49 ± 25.67 ng/ml for 28-day mortality). However, in the subgroup analysis, RD affected the predictive value of serum CC16 for 7-day mortality among the AKI group, CKD group, ARDS+AKI group, and ARDS+CKD group (Table 3).

**Table 3:**
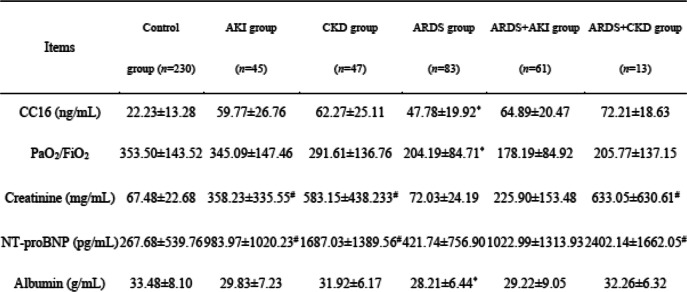
Serum CC16 level and other clinical parameters in the six groups

Notes: *CC16* Club cell protein 16, *NT-proBNP* N-terminal of the prohormone brain natriuretic peptide, *AKI* Acute kidney injury, *CKD* Chronic kidney disease, *ARDS* Acute respiratory distress syndrome

*P < 0.05 between the ARDS group and control group; ^#^*P* < 0.05 for the AKI group, CKD group, ARDS+AKI group, and ARDS+CKD group compared with the control group or ARDS group

## Discussion

According to previous research by our group, increased serum CC16 levels can help clinicians identify ARDS in critical care patients with normal renal function [10], and this finding was confirmed in the present study. However, RD, whether in the form of AKI or CKD, also was found to raise the serum CC16 level, reducing the specificity of serum CC16 for ARDS and altering the diagnostic cutoff points for ARDS. Additionally, serum CC16 no longer offered predictive value for survival outcome at 7 days in patients who had RD.

An increase in the serum CC16 concentration depends not only on increased transportation of the protein from the bronchoalveolar lavage fluid but also on decrease clearance of the protein in the kidney. Several previous studies have explored the value of the serum CC16 level for monitoring the permeability of the blood–air barrier, which shows a 1000-fold concentration gradient from bronchoalveolar lavage fluid (0.5–1.5 mg/L) to serum (10–15 ng/ml) in healthy nonsmokers. Therefore, increased CC16 levels have been reported in patents with multiple etiologies, such as chronic exposure to toxicants or severe air pollution [13–15]. However, CC16 has not been shown to be responsively synthesized, as evidenced by the negative correlation between serum CC16 and albumin levels in this study and previous research showing that the synthesis of albumin in response to ARDS can be rapidly reduced until the vascular compartment is repaired [16]. Furthermore, rapid renal clearance of serum CC16 with a half-life of approximately 2–3 h was previously found to occur via cubilin and/or megalin receptor-mediated endocytosis in the proximal tubule epithelial cells [17]. Andersson et al. showed that excretion of CC16 is related to the severity of renal damage, as measured by acute dimercaptosuccinic acid scintigraphy. Another clinical study indicated that the serum CC16 level may be able to predict creatinine clearance [18]. In addition, in vivo experiments have shown a significant increase in the serum CC16 level by 400-fold over the basal value after paraquat-induced lung injury [19]; in that study, the increase in serum CC16 was mainly determined by the degree of renal impairment. Therefore, according to the literature, two conditions will increase the serum CC16 concentration in critical care patients: reduced permeability of the blood–air barrier and dysfunction of renal clearance [20].

Decreased renal function has been proven previously to be associated with an elevated NT-proBNP level [21]. In the present study, a worsening renal condition, whether AKI, CKD, ARDS+AKI, or ARDS+CKD, synchronously raised not only the serum CC16 concentration but also the NT-proBNP and creatinine concentrations. The positive relationship between these renal conditions and the CC16, creatinine, and NT-proBNP levels reflected a higher prevalence of cardiac pulmonary edema in the control group in a study by Kropski et al [8]*,* which also observed baseline characteristics of cardiac pulmonary edema in their CKD and ARDS+CKD groups. Our analysis demonstrated that presentation of RD weakened the specificity and altered the cutoff value of serum CC16 for the diagnosis of ARDS, which may explain the conflicting results reported in the previous literature.

Although the serum CC16 level was not found to be associated with a 1-month clinical respiratory prognosis in a previous large, randomized trial investigation (*n* = 1200) [22], we found that an elevated serum CC16 level still predicted a poor outcome within 28 days in ICU patients with normal renal function. However, for patients with renal failure, this predictive value was lost within the first 7 days but regained by 28 days. Therefore, we propose that serum CC16 is still a useful biomarker for the diagnosis of and prognosis of ARDS in patients with normal renal function, especially given our previous finding that a reduced CC16 level can help clinicians predict the success of noninvasive ventilation among ICU patients [23]. Additionally, a decrease in the serum CC16 concentration might suggest a good prognosis, as this could be a sign of repair on the alveolar–capillary barrier in critical care patients. However, further studies are needed to prove this hypothesis.

Moreover, the biological role of an increased serum CC16 level in the ARDS process remains unclear. As an immunoregulatory protein, CC16 executes an anti-inflammatory function by inhibiting phospholipase A2 activation and promoting the expression of inflammatory cascades (interleukin [IL]-1b, IL-8, etc.), TH2 cell differentiation, and the migration of neutrophils and monocytes [24, 25]. Consistent with these findings, Pang et al. demonstrated that recombinant rat CC16 protein inhibits LPS-induced matrix metalloproteinase 9 expression and the production of pro-inflammatory cytokines via the nuclear factor-κB pathway in a model of tracheal epithelial cells and RAW264.7 macrophages [26, 27]. Those studies suggested that exogenous supplementation of recombinant CC16 could ameliorate cigarette smoke-induced lung inflammation in a murine disease model of chronic obstructive pulmonary disease [28]. Zhou et al. suggested that CC16 is an inhibitor of cell pyroptosis and inflammatory factors induced by LPS [29], demonstrating the potential value of CC16 in therapeutic efforts to repair acute lung injury induced by coronavirus disease 19 (COVID-19). Further investigations of the function of CC16 in critical care patients are needed in the future.

A few limitations of the present study should be noted. First, as a comprehensive ICU, we enrolled only critical care patients and no healthy patients who may have received interventions potentially affecting the serum CC16 level. Although previous studies have revealed a relatively stable median CC16 level in normal controls (5–7 ng/ml), the median CC16 value in critical care patients in our control group appeared to be higher (22.23 ± 13.28 ng/ml), likely because clinical conditions such as mechanical ventilation or primary graft dysfunction may promote the production of CC16 [30, 31]. This could have led to an unavoidable selection bias. However, the selection of ICU patients rather than healthy individuals as the control group for comparison is clinically meaningful for accurately diagnosing ARDS in the ICU. Second, we only monitored serum CC16 levels on admission, and the study lacked a parallel comparison with other promising biomarkers. Additional clinical research comparing the value of CC16 with that of other biomarkers for critical care patients is warranted with a broader range of patients. Thus, the exact diagnostic and prognostic roles of CC16 in ARDS require further investigation in a prospective study with a large sample size.

## Conclusion

The present study confirmed that an increased serum CC16 level can help identify ARDS and predict the outcome in critical care patients with normal renal function. However, RD, in the form of either AKI or CKD, also raises the serum CC16 concentration, which reduces the specificity of CC16 for ARDS in these patients and alters the optimal cutoff points. Moreover, the predictive value of CC16 for the survival outcome in ARDS patients with RD is lost before 7 days but regained by 28 days.

## Supplementary information

**Additional file 1: Supplementary Table 1** Reasons for intensive care unit (ICU) admission among patients in each group. Notes: AKI, acute kidney injury; CKD, chronic kidney disease; ARDS, acute respiratory distress syndrome; CPE, cardiogenic pulmonary edema. **Supplementary Table 2** Outcomes among intensive care unit (ICU) patients in each group. Notes: CRRT, continuous renal replacement therapy; NS, not significant. ^1^within 48 h after admission. **Supplementary Table 3.** Serum CC16 levels in different stage of AKI. Notes: CC16, club cell protein 16; AKI, acute kidney injury. **Supplementary Table 4.** Serum CC16 levels in different stage of CKD. Notes: CC16, club cell protein 16; CKD, chronic kidney disease.

## Data Availability

The data sets used and/or analyzed during the current study are available from the corresponding author on reasonable request.

## Abbreviations

AKI: Acute kidney injury
ARDS: Acute respiratory distress syndrome
CC16: Club cell protein 16
CKD: Chronic kidney disease
ICU: Intensive care unit
IL: Interleukin
LPS: Lipopolysaccharide
NT-proBNP: N-Terminal prohormone brain natriuretic peptide
RD: Renal dysfunction
ROC: Receiver operating characteristic curve
SBP: Systolic blood pressure
