# Correction to: Renal dysfunction reduces the diagnostic and prognostic value of serum CC16 for acute respiratory distress syndrome in intensive care patients

**DOI:** 10.1186/s12890-021-01417-6

**Published:** 2021-02-09

**Authors:** Jinle Lin, Wuyuan Tao, Jian Wei, Jian Wu, Wenwu Zhang, Jianbing Ye, Xuan Fu, Shiyong Zeng, Qingli Dou, Lijun Wang, Fang Tian

**Affiliations:** 1 Department of Emergency Medicine, Affiliated Baoan Hospital of Shenzhen, Southern Medical University, 118 LongjingEr Road, Baoan, Shenzhen, 518101 Guangdong China; 2 Department of Respiratory, East Zone Sixth Division, Guangdong Provincial People’s Hospital, Guangdong Academy of Medical Science, Guangdong Provincial Geriatrics Institute, The second School of Clinical Medicine, Southern Medical University, No. 106, Zhongshan Second Road, Guangzhou, 510000 Guangdong China; 3 Department of Critical Care Medicine, Affiliated Baoan Hospital of Shenzhen, Southern Medical University, Shenzhen, 518101 Guangdong China

## Correction to: BMC Pulm Med 20, 212 (2020) https://doi.org/10.1186/s12890-020-01245-0

Following publication of the original article [1], it came to the authors’ attention that the affiliations had been incorrectly numbered.

The numbering has now been corrected in the original article.

Please also find the corrected numbering in this correction.

The publisher apologizes for any inconvenience caused.
