# Supplementary material for: Renal dysfunction reduces the diagnostic and prognostic value of serum CC16 for acute respiratory distress syndrome in intensive care patients
Source: BMC Pulm Med. 2020 Aug 12;20:212. doi: 10.1186/s12890-020-01245-0 (PMC7422465; doi:10.1186/s12890-020-01245-0)
Supplement: Supplementary file 1 — Additional file 1: Supplementary Table 1 Reasons for intensive care unit (ICU) admission among patients in each group. Notes: AKI, acute kidney injury; CKD, chronic kidney disease; ARDS, acute respiratory distress syndrome; CPE, cardiogenic pulmonary edema. Supplementary Table 2 Outcomes among intensive care unit (ICU) patients in each group. Notes: CRRT, continuous renal replacement therapy; NS, not significant. 1within 48 h after admission. Supplementary Table 3. Serum CC16 levels in different stage of AKI. Notes: CC16, club cell protein 16; AKI, acute kidney injury. Supplementary Table 4. Serum CC16 levels in different stage of CKD. Notes: CC16, club cell protein 16; CKD, chronic kidney disease. [file 12890_2020_1245_MOESM1_ESM.docx]

**Supplementary table 1.** The reasons for intensive care unit (ICU) admission among patients in each group

| Items | Control group (*n*=230) | AKI group  (*n*=45) | CKD group  (*n*=47) | ARDS group  (*n*=83) | ARDS+AKI group  (*n*=61) | ARDS+CKD group  (*n*=13) |
| --- | --- | --- | --- | --- | --- | --- |
| Post-resuscitation, *n* (%) | 10(4.3) | 4(8.9) | 5(10.6) | 3(3.6) | 11(18) | 1(7.7) |
| Multiple injuries, *n* (%) | 16(7.0) | 2(4.5) | 0(0) | 6(7.2) | 2(3.3) | 0(0) |
| Neurological diseases, *n* (%) | 77(33.5) | 8(17.8) | 8(17.0) | 16(19.3) | 8(13.1) | 2(15.4) |
| Post-surgery, *n* (%) | 89(38.7) | 10(22.7) | 3(6.4) | 24(28.9) | 20(32.8) | 2(15.4) |
| Poisoning, *n* (%) | 9(3.9) | 2(4.9) | 0(0) | 1(1.3) | 2(3.6) | 0(0) |
| Pneumonia, *n* (%) | 57 (24.8) | 7(15.6) | 13 (27.7) | 71 (85.5) | 45 (73.8) | 12 (92.3) |
| Sepsis, *n* (%) | 14 (6.1) | 11 (24.4) | 5 (10.6) | 18 (21.7) | 26(42.6) | 3 (23.1) |
| CPE, *n* (%) | 4 (1.7) | 4 (8.9) | 13 (27.7)^#^ | 2 (2.4) | 5 (8.2) | 7 (53.8) |
| Pulmonary embolism, *n* (%) | 9(3.9) | 0(0) | 0(0) | 1(1.6) | 0(0) | 0(0) |
| Pulmonary tuberculosis, *n* (%) | 7(3.0) | 1(2.2) | 1(2.1) | 5(6.0) | 0(0) | 0(0) |
| Hyperthyroidism crisis, *n* (%) | 5(3.6) | 0(0) | 1(3.6) | 0(0) | 0(0) | 0(0) |
| Urinary infection, *n* (%) | 5(3.6) | 6(17.6) | 2(7.4) | 1(1.4) | 5(10.0) | 0(0) |
| Chronic lung disease, *n* (%) | 35(15.2) | 2(4.4) | 5(10.6) | 8(9.6) | 4(6.6) | 3(23.1) |
| Hemorrhagic diseases, *n* (%) | 10(5.9) | 7(17.5) | 0(0) | 3(3.8) | 4(7.3) | 1(8.3) |
| Obstetric diseases, *n* (%) | 19(8.3) | 1(2.2) | 0(0) | 6(7.2) | 2(3.3) | 0(0) |
| Inhalation injury, *n* (%) | 5(2.2) | 0(0) | 0(0) | 3(3.8) | 0(0) | 0(0) |
| Severe pulmonary Contusion, *n* (%) | 19(8.3) | 2(4.4) | 0(0) | 6(7.2) | 2(3.3) | 0(0) |
| Severe acute pancreatitis, *n* (%) | 9(3.9) | 4(8.9) | 0(0) | 3(3.6) | 7(11.5) | 0(0) |

Notes: AKI, acute kidney injury; CKD, chronic kidney disease; ARDS, acute respiratory distress syndrome; CPE, cardiogenic pulmonary edema.

**Supplementary table 2.** The outcomes of intensive care unit (ICU) patients in each group

| Items | Control group (*n*=230) | AKI group  (*n*=45) | CKD group  (*n*=47) | ARDS group  (*n*=83) | ARDS+AKI group  (*n*=61) | ARDS+CKD group  (*n*=13) |
| --- | --- | --- | --- | --- | --- | --- |
| Time of ventilation, days | 4.89±9.13 | 4.93±6.47 | 8.16±9.48 | 7.91±7.10 | 7.42±7.13 | 3.83±4.26 |
| Length of ICU stay, days | 10.62±14.73 | 8.73±7.80 | 12.15±11.92 | 10.89±9.15 | 12.17±9.94 | 7.22±4.29 |
| Length of hospital stay, daysays | 25.71±53.29 | 16.67±15.53 | 20.76±13.09 | 26.34±40.79 | 25.01±25.51 | 23.55±26.22 |
| The use of CRRT^1^,  *n* (%) | 6(2.60) | 26(57.77) | 32(68.08) | 6(7.22) | 26(42.62) | 9(69.23) |
| 7-day mortality, *n* (%) | 18(7.82) | 9(20.0) | 5(10.63) | 17(20.48) | 17(27.86) | 2(15.38) |
| 28-day mortality, *n* (%) | 32(13.91) | 15(33.33) | 11(23.40) | 33(39.75) | 27(44.26) | 3(23.07) |

Notes: CRRT, continuous renal replacement therapy; NS, not significant. ^1^within 48 h after admission；Any enrolled ICU patient who needed renal function support received CRRT.

**Supplementary Table 3.** Serum CC16 levels in different stage of AKI

| Items | Stage 1 in AKI group(n=33) | Stage 2 in AKI group(n=24) | Stage 3 in AKI group(n=49) |
| --- | --- | --- | --- |
| CC16 (ng/mL) | 59.76±20.56 | 64.43±25.99 | 63.86±24.08 |

Notes: CC16, club cell protein 16; AKI, acute kidney injury.

**Supplementary Table 4.** Serum CC16 levels in different stage of CKD

| Items | Stage 2 in CKD group(n=3) | Stage 3 in CKD group(n=10) | Stage 4 in CKD group(n=12) | Stage 5 in CKD group(n=35) |
| --- | --- | --- | --- | --- |
| CC16 (ng/mL) | 47.25±28.81 | 59.33±24.20 | 65.18±36.15 | 67.09±18.38 |

Notes: CC16, club cell protein 16; CKD, chronic kidney disease.
